# Clinicopathological characteristics, evolution, treatment pattern and outcomes of hormone-receptor-positive/HER2-low metastatic breast cancer

**DOI:** 10.3389/fendo.2023.1270453

**Published:** 2023-10-10

**Authors:** Shuhui You, Chengcheng Gong, Yi Li, Yizhao Xie, Yumeng Li, Yannan Zhao, Biyun Wang

**Affiliations:** ^1^ Department of Breast and Urological Medical Oncology, Fudan University Shanghai Cancer Center, Shanghai, China; ^2^ Department of Oncology, Shanghai Medical College, Fudan University, Shanghai, China; ^3^ Division of Hematology & Oncology, Department of Geriatrics, Second Affiliated Hospital, Guangzhou First People’s Hospital, College of Medicine, South China University of Technology, Guangzhou, Guangdong, China; ^4^ Department of Medical Oncology, Zhongshan Hospital Fudan University, Shanghai, China

**Keywords:** hormone receptor-positive, HER2-low, metastatic breast cancer, endocrine therapy, chemotherapy, evolution, prognosis

## Abstract

**Objective:**

Despite the promising efficacy of the novel antibody-drug conjugate trastuzumab deruxtecan in treating Hormone Receptor (HoR)-positive/Human Epidermal Growth Factor Receptor 2 (HER2)-low metastatic breast cancer (MBC), its categorization as a distinct entity remains disputed, as does the divergence in its endocrine and chemotherapy outcomes. This study aimed to elucidate the clinical characteristics, primary/metastatic lesion HER2 expression, and treatment outcomes of HoR-positive/HER2-low patients.

**Methods:**

We included HoR-positive/HER2-negative MBC patients who underwent 1^st^ and 2^nd^ line endocrine treatment from July 2010 to October 2022 at the Fudan University Shanghai Cancer Center, comparing the clinical pathological characteristics, HER2 expression in primary/metastatic lesions, treatment, and therapeutic effects of the HER2-low and HER2-zero groups.

**Results:**

Among the 458 HoR-positive/HER2-negative MBC patients, 54.37% (249/458) were HER2-low. The HER2-low group and the HER2-zero group had similar clinical pathological characteristics and similar progression-free survival (PFS) of 1^st^ and 2^nd^ line endocrine treatment (median PFS: 8.05 months vs 10.12 months, p=0.114, HR 1.257, 95% CI 0.771 to 1.028). The PFS of the HER2-low and HER2-zero groups was also similar, treated with different endocrine drugs (including aromatase inhibitors, tamoxifen/toremifene, fulvestrant, palbociclib, and everolimus). However, the HER2-low group had significantly shorter PFS during 1^st^ and 2^nd^ line chemotherapy compared to the HER2-zero group (median PFS: 8.64 vs 9.03 months, p=0.027, HR 0.841, 95% CI 0.721-0.980). Additionally, 41.18% (63/153) of patients exhibited a change in HER2 expression between primary and metastatic lesions. Notably, patients whose HER2 status changed from zero to low expression had significantly prolonged PFS during chemotherapy compared to those who maintained low HER2 expression (median PFS: 14.29 vs 11.27 months, p=0.048, HR 0.597, 95% CI 0.358-0.996).

**Conclusion:**

In HoR-positive MBC, patients with low and zero HER2 expression have similar clinical characteristics and respond similarly to endocrine treatment, but the chemotherapy effect is worse in the HER2-low patients. Moreover, the transformation of HER2 status from primary to metastatic lesions may have potential influence on chemotherapy outcomes. Therefore, the expression and heterogeneity of HER2 should be considered in clinical decisions.

## Introduction

Breast cancer (BC) is the leading cancer diagnosis among women globally (1), and its prevalence has increased over the last two decades (2). The transmembrane receptor tyrosine kinase Human Epidermal Growth Factor Receptor 2 (HER2) belongs to the epidermal growth factor receptor family. Breast cancers with immunohistochemistry (IHC) scores of 1+ or 2+ and negative *in situ* hybridization (ISH) are termed HER2-low according to the 2018 ASCO/CAP guidelines (3, 4). The reported prevalence of HER2-low breast cancer spans from 31.0% to 61.6% across various studies and breast cancer types (5).

The DESTINY-Breast04 trial spotlighted the superior efficacy of HER2-targeted antibody-drug conjugates (ADCs) compared to standard chemotherapy options in patients with HER2-low advanced breast cancer (6). This emphasizes the clinical significance of the HER2-low patient population, advocating for a redefinition of subgroups within HER2-negative breast cancers. Several studies have suggested distinctive clinicopathological characteristics between HER2-low and HER2-zero breast cancer patients (3, 7–9). Nonetheless, the unique clinical-pathologic attributes of HER2-low BC remain to be definitively established.

Recent evidence points to a higher prevalence of HER2-low in Hormone Receptor (HoR)-positive breast cancer compared to its HoR-negative counterpart (5, 10). From a biological perspective, this could be attributed to the co-expression of HoR (7, 11) and a low level of HER2 expression, which might be a therapeutic resistance factor (12) in HoR-positive BC due to the interplay between HoR-signaling and HER2 signaling. Although prior research has attempted to discern prognostic disparities between HER2-low and HER2-zero in HoR-positive metastatic breast cancer (MBC), results remain inconclusive (8, 13, 14). Additionally, the use of various systemic therapy regimens in these studies further complicates the evaluation of survival rates. Consequently, there is an urgent need for additional investigations into HoR-positive/HER2-low MBC.

In our study, we analyzed data from 458 patients with HoR-positive/HER2-negative MBC. Our focus was to illustrate the clinicopathological characteristics, evolution, treatment patterns, and the effectiveness of the 1st and 2nd line advanced systemic therapy in HoR-positive/HER2-low tumors, and to draw comparisons with HER2-zero tumors.

## Materials and methods

### Participants

Subjects were 458 patients with HoR-positive/HER2-negative recurrent or metastatic breast cancer who received endocrine therapy for advanced systemic treatment at the Fudan University Shanghai Cancer Center (FUSCC) between July 2010 and October 2022.

The eligibility criteria were (1) female gender; (2) age 18 years; (3) histologically and cytologically proven unresectable or metastatic breast cancer; (4) HoR positive and HER2 negative status according to the last IHC test; (5) at least one cycle of endocrine therapy (ET) during advanced systemic treatment; and (6) comprehensive medical records. Through a retrospective evaluation of medical records, clinical data including baseline patient characteristics, pathology reports, treatment history, and survival outcomes were acquired.

Patients were divided into HER2-low and HER2-zero groups according on their baseline pathological characteristics. HoR-positive is defined as ER expression ≥1%. Immunohistochemistry and/or *in situ* hybridization are utilized to evaluate the HER2 status. HER2 negativity is characterized by an IHC score of 0-1+ or IHC2+/ISH-negative (3). Low HER2 positivity is defined as a score of 1+ on IHC analysis or as a score of 2+ on IHC with negative ISH results, whereas HER2 zero expression is defined as an IHC score of 0. In cases where there was a discrepancy in HER2 status between the primary tumor and metastatic lesions, the HER2 status from the latest pathological result before the first-line advanced systemic treatment was employed. For patients with varying HER2 results across multiple metastatic site biopsies, we used the most dominant expression pattern. In instances where no dominant pattern was evident or discrepancies were equally distributed, the HER2 status derived from the biopsy of the largest metastatic site determined the categorization.

### Evaluation

If available, the pathology reports of the primary tumors and the recurrent/metastatic sites were obtained. The base line pathology was the latest pathology report, prior to the first-line endocrine therapy for MBC. The following baseline clinicopathological parameters were evaluated by HER2-low or HER2-zero status: age, sex, Eastern Cooperative Oncology Group (ECOG) Performance Status, menopausal status, tumor grade, nodal status, histological type, clinicopathologic stage at diagnosis, Ki-67, disease-free interval (DFI), number of metastatic sites, visceral metastases, metastatic sites, resistance status to endocrine therapy, Luminal subtype, and estrogen receptor (ER) expression.

Primary endocrine resistance to endocrine therapy (ET) is defined as relapse while on the first 2 years of adjuvant ET, or progressive disease (PD) within the first 6 months of first-line ET for MBC, while on ET. Secondary endocrine resistance is defined as relapse while on adjuvant ET but after the first 2 years, or relapse within 12 months of completing adjuvant ET, or PD ≥6 months after initiating ET for MBC, while on ET (15). Endocrine-sensitive patients were defined as patients who never received ET in early breast cancer stage, or relapsing ≥12 months after completing adjuvant ET, or diagnosed with *de novo* stage IV breast cancer (16).

Progression-free survival (PFS) of different groups (HER2-low and HER2-zero) was the outcome measurement; treatment safety were secondary measurements. PFS was defined as time from the start of administration to the progression of disease or the occurrence of death. The Common Terminology Criteria for Adverse Events (CTCAE) version 4.03 of the National Cancer Institute was used to evaluate safety. Complete response (CR), partial response (PR), stable disease (SD), and PD were evaluated using Response Evaluation Criteria in Solid Tumors (RECIST) version 1.1.

### Statistical analysis

Descriptive data included information on baseline clinicopathological parameters and treatment options. Baseline features were compared between groups (HER2-low vs. HER2-zero). Differences by HER2-low status were examined using the chi-square or Fisher’s exact tests for categorical variables and the Wilcoxon rank test for continuous data. The concordance between HER2 expression on primary tumors and matched recurrent/metastatic samples was assessed using Cohen’s kappa coefficient (K). The Kaplan-Meier method was used to estimate PFS. We applied the log-rank test to juxtapose the PFS of both groups and employed the Mantel-Haenszel method to compute hazard ratios (HRs) along with their 95% confidence intervals (CI). All p values were based on a two‐sided hypothesis. A p-value < 0.05 was considered significant. SPSS software (SPSS version 21.0, SPSS Inc., Chicago, IL) was used for statistical evaluations.

## Results

### Patients baseline characteristics

Our research, conducted from June 2010 to October 2022, included a total of 458 HoR-positive/HER2-negative MBC patients (**Table 1**). Of these, 54.37% (249/458) had HER2-low MBC, while the remaining 45.63% (209/458) were HER2-zero. The patients in the HER2-low group had a median age of 56 years (range: 28-85). There were no significant differences in the ECOG performance status between the two groups (p=0.486). Histological grade II and invasive ductal carcinoma of no specific type (IDC-NST) were comparably more common among HoR-positive/HER2-low individuals. Among these individuals, those with 1%-9% ER positivity constituted a minority at 5.17% (6/116), whereas the majority at 94.83% (110/116) had 10%-100% ER positivity. As for the number of metastatic lesions, 78.82% (361/458) of HER2-low patients had at least two. Furthermore, a substantial 75.5% (188/249) had visceral metastases. Looking into their treatment responsiveness, nearly half of the HoR-positive/HER2-low patients (49.00%, 122/249) demonstrated sensitivity to first-line endocrine treatment for MBC. However, primary endocrine resistance was present in 19.28% (48/249) of the cases. In general, demographics at baseline were fairly consistent across both the HER2-low and HER2-negative groups (**Table 1**).

**Table 1:** Baseline patient characteristics stratified by HER2 status (HER2-low vs. HER2-zero).

Demographics	Total N=458	HER2-low N=249	HER2-zero N=209	*p*-value
Age, Median(range)	55(28-85)	56(28-85)	54(28-79)	0.721
** <65 years**	375(81.9%)	199(79.9%)	176(84.2%)	0.235
** ≥65 years**	83(28.1%)	50(20.1%)	33(15.8%)	
ECOG performance status				0.486
** 0~1**	430(93.9%)	232(93.2%)	198(94.7%)	
** ≥2**	28(11.2%)	17(6.8%)	11(5.3%)	
Menopausal status				0.886
** Premenopausal**	111(24.2%)	61(24.5%)	50(23.9%)	
** Postmenopausal**	347(75.8%)	188(75.5%)	159(76.1%)	
Grade				0.348
** I**	3(0.7%)	0(0%)	3(1.4%)	
** II**	157(34.3%)	84(33.7%)	73(34.9%)	
** III**	101(22.1%)	56(22.5%)	45(21.5%)	
** Unknown**	197(43.0%)	109(43.8%)	88(42.1%)	
Histological type				0.195
** IDC-NST**	386(84.3%)	211(84.7%)	175(83.7%)	
** ILC**	19(4.2%)	11(4.4%)	8(3.8%)	
** Other**	21(4.6%)	7(2.8%)	14(5.6%)	
** Unknown**	32(7.0%)	20(8.0%)	12(4.8%)	
Stage at diagnosis				0.412
** I**	52(11.4%)	26(20.4%)	26(12.4%)	
** II**	81(17.7%)	45(18.1%)	36(17.2%)	
** III**	182(39.7%)	98(39.4%)	84(40.2%)	
** IV**	48(10.5%)	32(12.9%)	16(7.7%)	
** Unknown**	95(20.7%)	48(19.3%)	47(22.5%)	
Ki-67				
** Median ± SD**	27.42 ± 1.22	28.91 ± 1.68	25.51 ± 1.76	0.168
** <20%**	115(25.1%)	57(22.9%)	58(27.8%)	0.063
** ≥20%**	168(36.7%)	102(41.0%)	66(31.6%)	
** Unknown**	175(38.2%)	90(36.1%)	85(40.7%)	
DFI^b^				0.103
** DFI<2 years**	95(29.7%)	54(21.7%)	41(19.6%)	
** DFI≥2 years**	313(68.3%)	162(65.1%)	151(72.2%)	
** *De novo* stage IV**	49(10.7%)	32(12.9%)	16(7.7%)	
** Unknown**	1(0.2%)	0(0.0%)	1(0.5%)	
Number of metastatic sites^c^				0.188
** 1**	97	47	50	
** ≥2**	361	202	159	
Visceral metastases				0.214
** Yes**	335(73.1%)	188(75.5%)	147(70.3%)	
** No**	123(26.9%)	61(24.5%)	62(29.7%)	
Metastatic sites				
** Lung**	261(57.0%)	139(30.3%)	122(58.4%)	0.583
** Liver**	198(43.2%)	110(24.0%)	88(42.1%)	0.656
** Brain**	21(4.6%)	13(2.8%)	8(3.8%)	0.485
** Bone alone**	34(7.4%)	15(6.0%)	19(9.1%)	0.212
Resistance status to ET^d^				0.799
** Primary resistance**	84(18.3%)	48(19.3%)	36(17.2%)	
** Secondary resistance**	139(30.3%)	74(29.7%)	65(31.1%)	
** Sensitivity**	230(50.2%)	122(49.0%)	108(51.7%)	
Subtype				0.554
** Luminal A**	131(28.6%)	72(28.9%)	59(28.2%)	
** Luminal B**	246(53.7%)	143(57.4%)	103(49.3%)	
** Unknown**	81(17.7%)	34(13.7%)	47(22.5%)	
ER expression				0.333
** ≥10%**	189(41.3%)	110(44.2%)	79(31.7%)	
** 1-9%**	10(2.2%)	6(2.4%)	4(1.9%)	
** Unknown**	259(56.6%)	133(53.4%)	126(60.3%)	

a. IDC-NST, invasive ductal carcinoma no specific type; ILC, invasive lobular carcinoma.

b. Disease‐free interval (DFI) is defined as the time from diagnosis of breast cancer to first relapse.

c. Data of Metastases were collected at the time before the 1^st^ line endocrine therapy for MBC.

d. Primary endocrine resistance is defined as relapse while on the first 2 years of adjuvant ET, or PD within the first 6 months of first-line ET for ABC, while on ET. Secondary endocrine resistance is defined as relapse while on adjuvant ET but after the first 2 years, or relapse within 12 months of completing adjuvant ET, or PD ≥6 months after initiating ET for ABC, while on ET (15)^(p5)^.

### Evolution of HER2 status from primary to recurrent/metastatic BC

**Figure 1:**
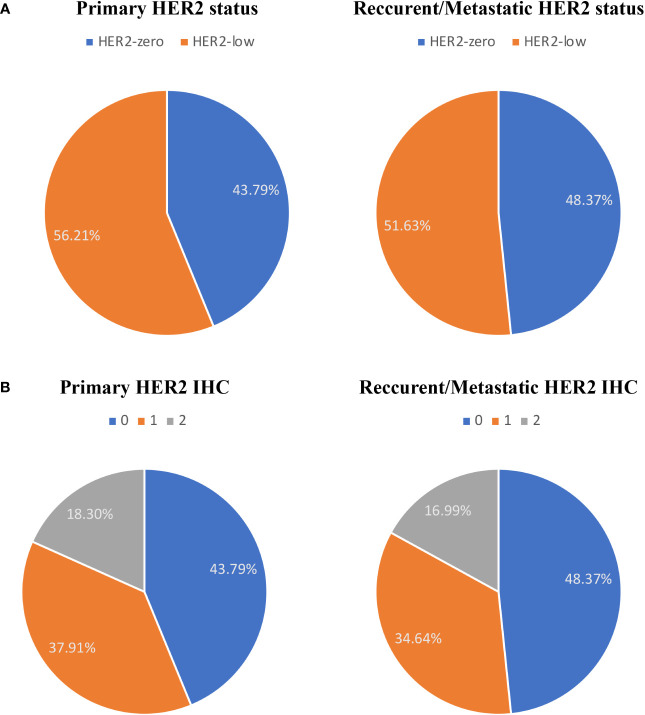
The compositions of human epidermal growth factor receptor 2 (HER2)-negative population by **(A)** HER2 status and **(B)** HER2 immunohistochemistry (IHC) score.

**Figure 2:**
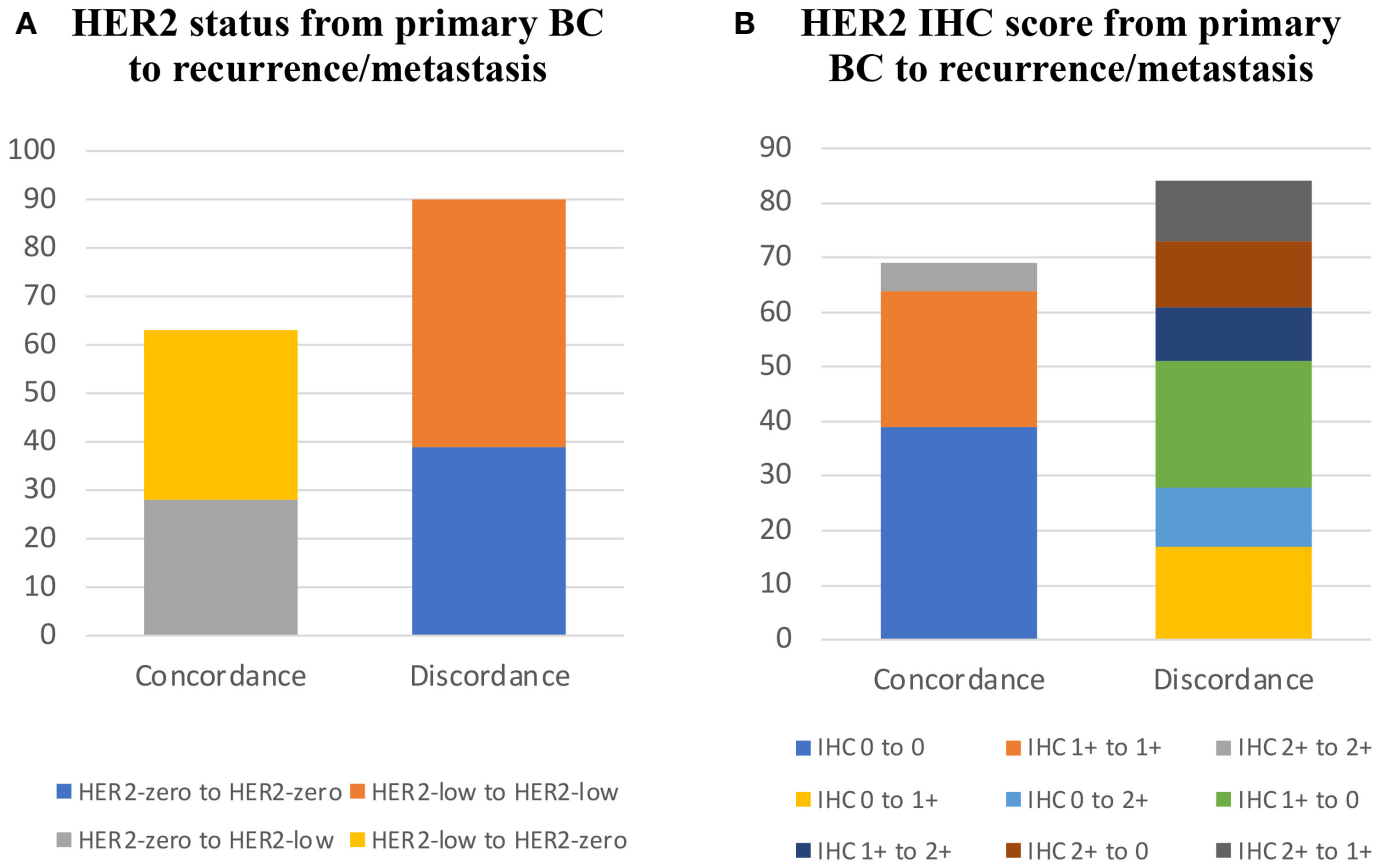
HER2 expression evolution from primary BC to recurrence/metastasis. **(A)** HER2 status from primary BC to recurrence/metastasis. **(B)** HER2 IHC score from primary BC to recurrence/metastasis.

Both primary and recurrent/metastatic IHC test findings were present in 153 individuals. When compared to the primary tumor, matched biopsy samples taken at the time of relapse showed a substantial difference in HER2-expression. 56.21% (86/153) of primary tumors were HER2-low, whereas 51.63% (79/153) of recurrent/metastatic tumors were HER2-low (**Figure 1A**, **Table 2**). The proportion of discordance between HER2 status in primary and metastatic tissue was 41.18% (63/153) (K=0.17, 95%CI 0.02-0.33) (**Figure 2A**, **Table 2**). 18.30% (28/153) of patients were converted from HER2-zero to HER2-low, whereas an opposite trend (from HER2-low to HER2-zero) was observed in 22.88% (35/153) patients (**Table 2**). 54.90% (84/153) of HER2 expression was inconsistent according to the HER2 IHC score (K = 0.12, 95%CI 0.00-0.24) (**Figure 2B**, **Table 3**). The proportion of HER2 IHC1+ patients decreased from 37.91% (58/153) in the primary tumor to 34.64% (53/153) in the recurrence/metastasis (**Figure 1B**, **Table 3**).

**Table 2:** HER2 expression evolution from primary BC to recurrence/metastasis according to HER2 status (HER2-low vs. HER2-zero).

		Recurrence/Metastasis N (%)		Total
		HER2-zero	HER2-low	
Primary BC N (%)	HER2-zero	39(25.49)	28(18.30)	67(43.79)
	HER2-low	35(22.88)	51(33.33)	86(56.21)
Total		74(48.37)	79(51.63)	153(100)
HER2 status discordance rate=41.18% (K=0.17, 95%CI 0.02-0.33)				

BC, breast cancer; N, number.

**Table 3:** HER2 expression evolution from primary BC to recurrence/metastasis according to HER2 IHC score (HER2 0 vs. HER2 1+ vs. HER2 2+).

		Recurrence/metastasis N (%)			Total
		HER2 0	HER2 1+	HER2 2+	
Primary BC N (%)	HER2 0	39(25.49)	17(11.11)	11(7.19)	67(43.79)
	HER2 1+	23(15.03)	25(16.34)	10(6.54)	58(37.91)
	HER2 2+	12(7.84)	11(7.19)	5(3.27)	28(18.30)
Total		74(48.37)	53(34.64)	26(16.99)	153(100)
HER2 IHC score discordance rate=54.90% (K = 0.12, 95%CI 0.00-0.24)					

BC, breast cancer; N, number.

### Treatment and efficacy

#### Endocrine therapy

As of October 2022, all patients had commenced first-line endocrine therapy for MBC, with 94.10% (431/458) showing documented disease progression. Second-line endocrine therapy was administered to 83.84% (384/458) of the patients, 70.09% (321/458) of whom exhibited tumor progression. When comparing treatment patterns between HER2-low and HER2-negative populations, no significant differences were identified (**Table 4**).

**Table 4:** Treatment patterns in the 1^st^-2^nd^ line settings of HoR-positive MBC stratified by HER2 status (HER2-low vs. HER2-zero).

Treatment patterns	Total N=458	HER2-low N=249	HER2-zero N=209	*p*-value
Chemotherapy as the first-line advanced systematic therapy				0.653
Yes	103(22.5%)	58(23.3%)	45(23.5%)	
No	355(77.5%)	191(76.7%)	164(78.5%)	
First-line endocrine therapy^a^				0.566
TOM/TOR	34(7.4%)	23(9.2%)	11(5.3%)	
AI	290(63.3%)	154(61.8%)	136(65.1%)	
FUL	72(15.7%)	36(14.5%)	36(17.2%)	
AI +FUL	11(2.4%)	8(3.2%)	3(1.4%)	
AI+CDK4/6i	19(4.1%)	10(4.0%)	9(4.3%)	
FUL+CDK4/6i	24(5.2%)	14(5.6%)	10(4.8%)	
Endocrine therapy only	318(69.4%)	178(71.5%)	140(67.0%)	0.298
Second-line endocrine therapy^b^				0.337
TOM/TOR	21(5.5%)	13(6.2%)	8(4.6%)	
AI	175(45.7%)	89(42.4%)	87(50.3%)	
AI+FUL	10(2.6%)	5(2.4%)	5(2.9%)	
FUL	120(31.3%)	74(35.2%)	46(26.6%)	
FUL+CDK4/6i	26(6.8%)	14(6.7%)	12(6.9%)	
AI+CDK4/6i	26(6.8%)	11(5.2%)	15(8.7%)	
Megastrol	2(0.5%)	2(1.0%)	0(0%)	
Endocrine therapy only	318(83.0%)	172(81.9%)	146(84.4%)	0.604
First-line chemotherapy				0.384
Taxane^c^	59(14.1%)	30 (13.2%)	29(15.3%)	
Taxane + platinum	50(12.0%)	22(9.6%)	28(14.8%)	
Taxane + gemcitabine	60(14.4%)	33(14.5%)	27(14.3%)	
Taxane + anthraquinone+ cyclophosphamide	12(2.9%)	6(2.6%)	4(2.1%)	
Taxane + capecitabine	39(9.4%)	22(9.6%)	17(9.0%)	
Capecitabine	105(25.2%)	56(24.6%)	49(25.9%)	
Vinorelbine	14(3.4%)	12(5.3%)	2(1.1%)	
Vinorelbine + others	7(1.7%)	4(1.8%)	3(1.6%)	
Capecitabine + vinorelbine	38(4.1%)	23(10.1%)	15(7.9%)	
Gemcitabine + platinum	4(1.0%)	1(0.4%)	3(1.6%)	
Anthraquinone + cyclophosphamide	16(3.8%)	12(5.3%)	5(2.6%)	
FOLFOX	3(0.7%)	1(0.4%)	2(1.1%)	
Others	10(2.4%)	5(2.2%)	5(2.6%)	
Chemotherapy with taxane	220(52.8%)	114(50.5%)	106(56.1%)	0.215
Chemotherapy with capecitabine^d^	211(50.6%)	121(53.1%)	90(47.6%)	0.268
Second-line chemotherapy				0.473
Taxane	49(14.4%)	33(17.6%)	16(10.5%)	
Taxane + platinum	19(5.6%)	13(6.9%)	6(3.9%)	
Taxane + gemcitabine	69(20.2%)	31(16.5%)	38(24.8%)	
Taxane + anthraquinone + cyclophosphamide	3(0.9%)	2(1.1%)	1(0.7%)	
Taxane + capecitabine	7(2.1%)	4(2.1%)	3(2.0%)	
Capecitabine	72(21.1%)	37(19.7%)	35(22.9%)	
Vinorelbine	36(10.6%)	20(10.6%)	16(10.5%)	
Vinorelbine + others	7(2.1%)	5(2.7%)	2(1.3%)	
Capecitabine + vinorelbine	29(8.5%)	14(7.4%)	15(9.8%)	
Gemcitabine + platinum	9(2.6%)	5(2.7%)	4(2.6%)	
Anthraquinone + cyclophosphamide	5(1.5%)	4(2.1%)	1(0.7%)	
Eribulin	9(2.6%)	5(2.7%)	4(2.6%)	
VP-16	6(1.8%)	1(0.5%)	5(3.3%)	
FOLFOX	5(1.5%)	3(1.6%)	2(1.3%)	
Others	16(4.7%)	11(5.9%)	5(3.3%)	
Chemotherapy with taxane	150(44.0%)	85(45.2%)	65(42.5%)	0.614
Chemotherapy with capecitabine	124(37.2%)	67(35.6%)	57(37.3%)	0.758

a. Eight patients (4 HER2-low and 4 HER2-zero) had received AI and TAM/TOR successively at 1st line endocrine therapy for MBC.

b. 383 patients had second-line endocrine therapy, including 210 HER2-low patients and 173 HER2-zero patients. There was one HER2-low patient who received changed AI to TOM/TOR at 2nd line endocrine therapy, another HER2-low patient receiving both TOR and CDK46i at 2nd line endocrine therapy.

c. Taxanes represent a category of drugs within the paclitaxel family, including docetaxel, liposomal paclitaxel, and albumin-bound paclitaxel. Chemotherapy with capecitabine includes treatments intended for maintenance therapy purposes.

d. AI, aromatase inhibitors; TAM, tamoxifen; TOR, toremifene; FUL, fulvestrant; CDK46i, inhibitors to CDK4/6; FOLFOX, folinic acid + fluorouracil + oxaliplatin.

**Figure 3:**
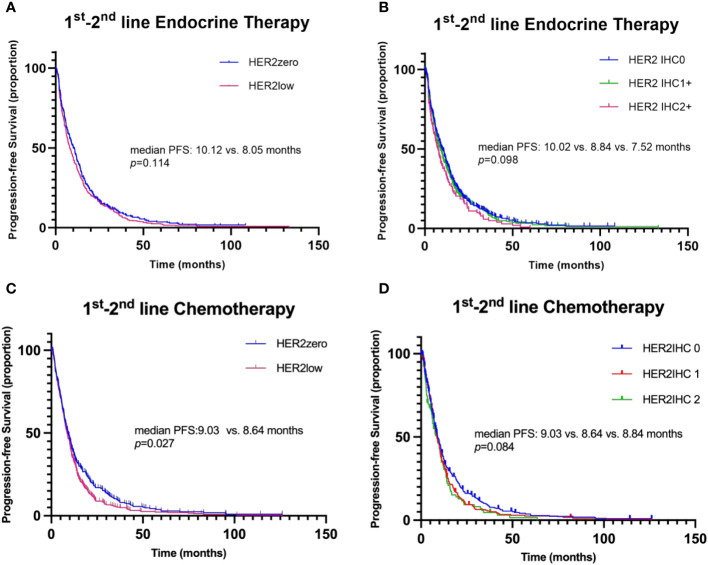
Kaplan–Meier curves for progression-free survival (PFS) in the 1^st^-2^nd^ line systemic therapy. **(A)** Comparison of PFS between patients with HER2-zero and HER2-low status during 1^st^-2^nd^ line endocrine therapy. **(B)** Comparison of PFS among patients with HER2 IHC scores of 0, 1+, and 2+ during 1^st^-2^nd^ line endocrine therapy. **(C)** Comparison of PFS between patients with HER2-zero and HER2-low status during 1^st^-2^nd^ line chemotherapy. **(D)** Comparison of PFS among patients with HER2 IHC scores of 0, 1+, and 2+ during 1^st^-2^nd^ line chemotherapy.

Notably, the Kaplan-Meier analysis suggested no significant distinction in PFS between HER2-zero and HER2-low patients undergoing 1st-2nd line endocrine therapy (median PFS: 10.12 vs. 8.05 months, p=0.114, HR 1.257, 95% CI 0.771 to 1.028; **Figure 3A**). The same analysis also unveiled no marked difference in PFS amongst patients with varying HER2 IHC scores of 0, 1+, and 2+ within the same therapy period (median PFS: 10.02 vs. 8.84 vs. 7.52 months, p=0.098; **Figure 3B**).

Within the HoR-positive/HER2-low group, aromatase inhibitors (AI) were the most commonly employed first-line endocrine treatment, given to 61.8% (154/249) of patients, followed by fulvestrant (FUL), which was provided to 14.5% (36/429). Among HER2-low patients receiving second-line endocrine treatment, AI (42.4%, 89/210) and FUL (35.2%, 74/210) were the most frequently utilized.

**Figure 4:**
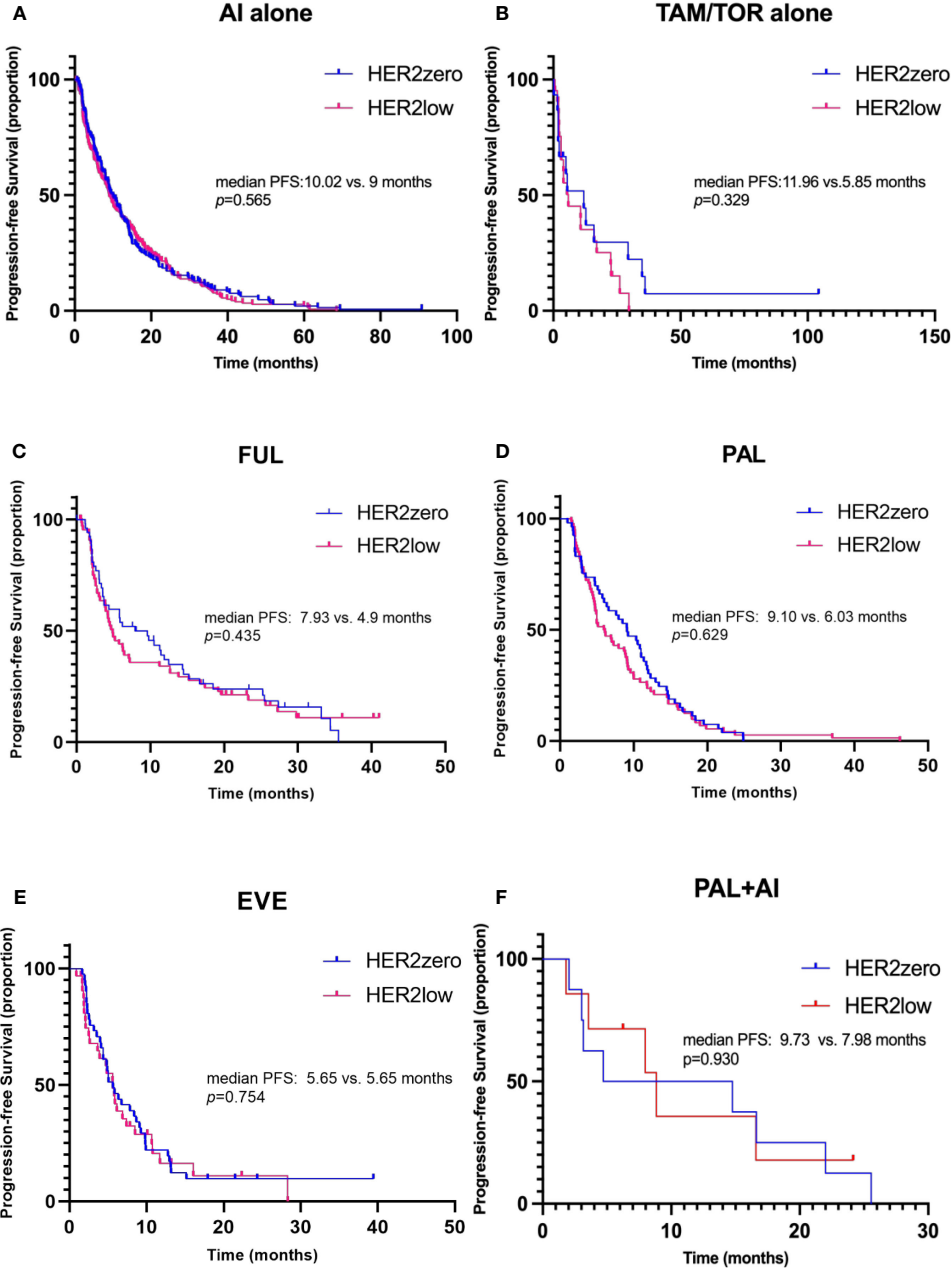
Kaplan–Meier curves of progression-free survival (PFS) during 1^st^-2^nd^ line endocrine therapy (ET), stratified by different treatment regimens. **(A)** PFS in HER2-zero versus HER2-low patients receiving aromatase inhibitors (AI) alone as 1^st^-2^nd^ line ET. **(B)** PFS in patients receiving tamoxifen/toremifene (TAM/TOR) alone. **(C)** PFS in patients receiving fulvestrant (FUL). **(D)** PFS in patients receiving palbociclib (PAL). **(E)** PFS in patients receiving everolimus (EVE). **(F)** PFS in patients receiving PAL combined with AI for the first-line therapy.

In patients receiving AI or tamoxifen (TAM)/toremifene (TOR) alone as first- or second-line endocrine treatment, no difference in PFS was found between HER2-zero and HER2-low groups (**Figures 4A, B**, median PFS: 10.02 vs. 9.00 months, p=0.565, HR 0.941, 95% CI 0.764-1.158; median PFS: 11.96 vs. 5.85 months, p=0.329, HR 0.693, 95% CI 0.332-1.447, respectively). Similarly, the PFS between HER2-zero and HER2-low patients receiving FUL, Palbociclib (PAL), or everolimus (EVE) as first- or second-line endocrine therapy also showed no significant difference (median PFS: 7.93 vs. 4.9 months, p=0.435, HR 0.930, 95% CI 0.623-1.389; median PFS: 9.10 vs. 6.03 months, p=0.629, HR 0.885, 95% CI 0.619-1.264; median PFS: 5.65 vs. 5.65 months, p=0.754, HR 0.923, 95% CI 0.559-1.524, respectively) (**Figures 4C–E**). Even though HER2-low status was associated with a shorter PFS in patients receiving endocrine treatment with FUL or PAL in the 1st-2nd line, the difference was not statistically significant. Moreover, for those treated with a combination of PAL and AI as a first-line therapy, both HER2-zero and HER2-low groups showed no significant PFS difference, with results indicating a median PFS of 9.73 months and 7.89 months, respectively (p=0.930, HR 1.053, 95% CI 0.330-3.357) (**Figure 4F**).

When patients were further categorized according to endocrine-resistant status, ER expression, and Luminal subtypes, no significant differences were discernible in the PFS of 1st-2nd line ET between HER2-zero and HER2-low patients, regardless of ET-sensitivity, ER expression range, Luminal types and menopausal status (**Supplementary Figure 2**).

Furthermore, variations in HER2 status from primary sites to the metastasis, whether concordant or discordant, showed no substantial impact on PFS during the 1st-2nd line of endocrine therapy (median PFS: 14.19 vs. 13.47 months, p=0.602, HR 1.093, 95% CI 0.782-1.527; **Figure 6A**).

#### Chemotherapy

Of the enrolled participants, 91.0% (417/458) underwent first-line chemotherapy for advanced disease, while 68.6% (341/458) received second-line chemotherapy. The selection of chemotherapy, whether in the first or second line, showed an even distribution between the HER2-low and HER2-zero subgroups of HoR-positive MBC patients (**Table 4**). Notably, capecitabine and taxane-based regimens were the dominant choices, and the decision to use these regimens wasn’t significantly swayed by the patient’s HER2 status. Diverging from the pattern observed in endocrine therapy, a significantly longer PFS was observed in HER2-zero patients compared to those with HER2-low status during the 1st-2nd line chemotherapy (median PFS: 9.03 vs. 8.64 months, p=0.027, HR 0.841, 95% CI 0.721-0.980; **Figure 3C**). In the context of 1st-2nd line chemotherapy, patients with varying HER2 IHC scores (0, 1+, and 2+) showed no significant difference in PFS (median PFS: 9.03 vs. 8.64 vs. 8.84 months, p=0.084; **Figure 3D**). In the multivariate analysis, HER2 status (HER2-zero vs. HER2-low) was also significantly associated with PFS during 1st-2nd line chemotherapy (HR 0.849, 95% CI 0.727-0.990, p=0.037; **Table 5**).

**Table 5:** Univariate and multivariate cox regression analysis of factors associated with progression-free survival of the first- or second-line chemotherapy.

Characteristic	Univariate analysis		Multivariate analysis	
	Hazard Ratio (95% CI)	p-value	Hazard Ratio (95% CI)	p-value
**Age group (≥65 years vs. <65 years)**	0.953 (0.772-1.176)	0.653		
**ECOG (0~1 vs. ≥2)**	0.682 (0.498-0.933)	0.017*	0.703 (0.513-0.963)	0.028*
**Menopausal status (postmenopausal vs. premenopausal)**	1.143 (0.959-1.363)	0.136		
**ER expression (<10% vs. ≥10%)**	0.619 (0.321-1.193)	0.152		
**Ki-67 (≤14% vs. >14%)**	0.884 (0.664-1.177)	0.399		
**HER2 status (HER2-zero vs. HER2-low)**	0.841 (0.721-0.981)	0.027*	0.849 (0.727-0.990)	0.037*
**Subtypes (Luminal A vs. Luminal B)**	0.941 (0.745-1.188)	0.609		
***De novo* stage IV (Yes vs. No)**	1.086 (0.847-1.392)	0.516		
**Number of metastatic sites (1 vs. ≥2)**	0.748 (0.609-0.918)	0.006*	0.764 (0.622-0.939)	0.010*
**Visceral metastases (No vs. Yes)**	0.859 (0.715-1.031)	0.103		

*p< 0.05.

**Figure 5:**
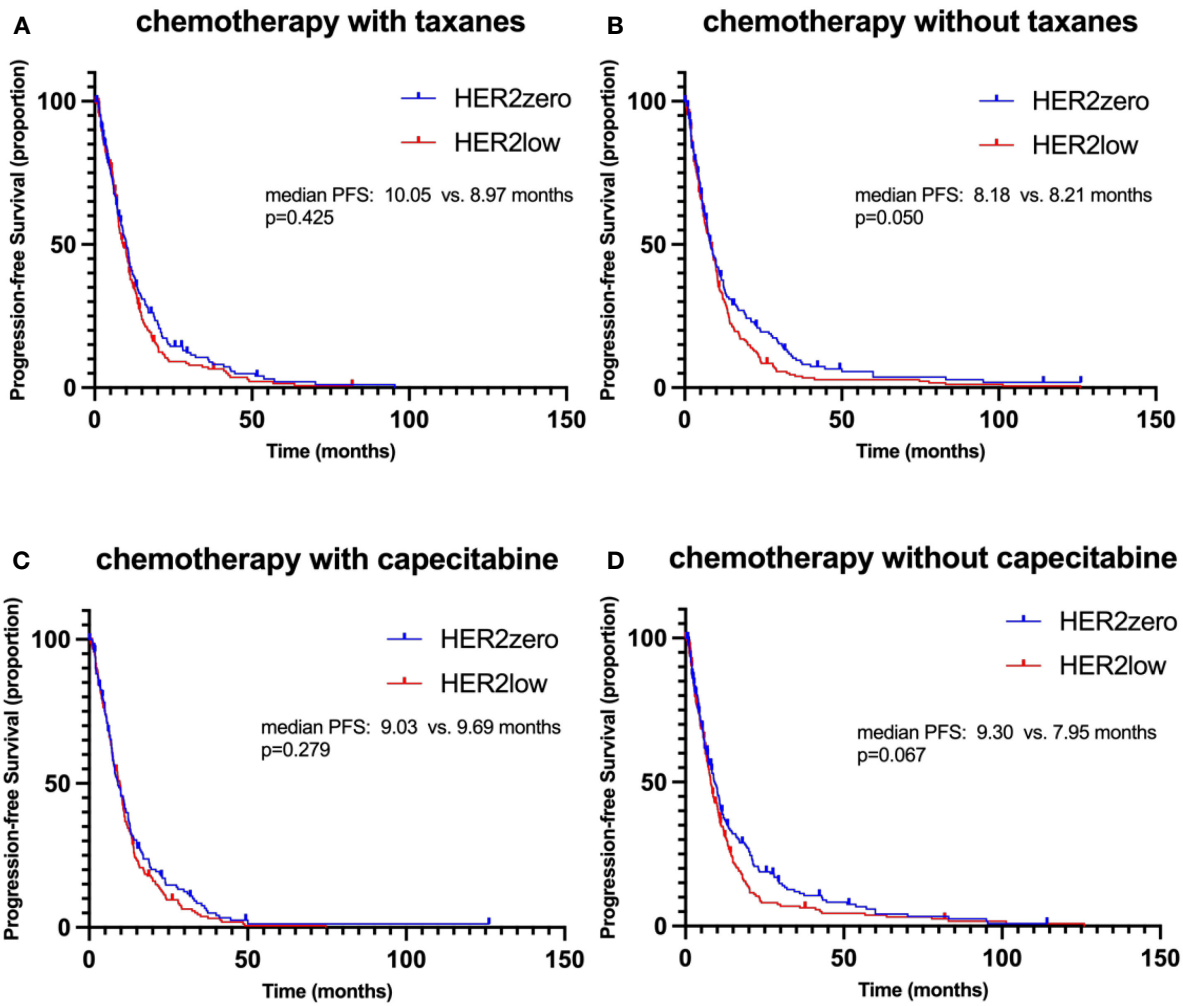
Kaplan–Meier curves of progression-free survival (PFS) during 1^st^-2^nd^ line chemotherapy, stratified by different treatment regimens. **(A)** PFS in HER2-zero versus HER2-low patients receiving chemotherapy with taxanes. **(B)** PFS in HER2-zero versus HER2-low patients receiving chemotherapy without taxanes. **(C)** PFS in HER2-zero versus HER2-low patients receiving chemotherapy with capecitabine. **(D)** PFS in HER2-zero versus HER2-low patients receiving chemotherapy without capecitabine.

In the taxane-based chemotherapy cohort, the median PFS was 10.05 months for HER2-zero patients and 8.97 months for the HER2-low group, a non-significant difference (p=0.425, HR 0.866, 95% CI 0.694-1.081; **Figure 5A**). Without taxane, both HER2-zero and HER2-low patients showed almost identical PFS of 8.18 and 8.21 months, nearing statistical significance (p=0.050, HR 0.807, 95% CI 0.652-1.000; **Figure 5B**). In the capecitabine-treated group, the HER2-zero and HER2-low patients had PFS of 9.03 and 9.69 months, respectively, without significant difference (p=0.279, HR 0.932, 95% CI 0.744-1.167; **Figure 5C**). Without capecitabine, the PFS was 9.30 months for HER2-zero and 7.95 months for HER2-low, a near-significant difference (p=0.067, HR 0.821, 95% CI 0.665-1.014, **Figure 5D**).

**Figure 6:**
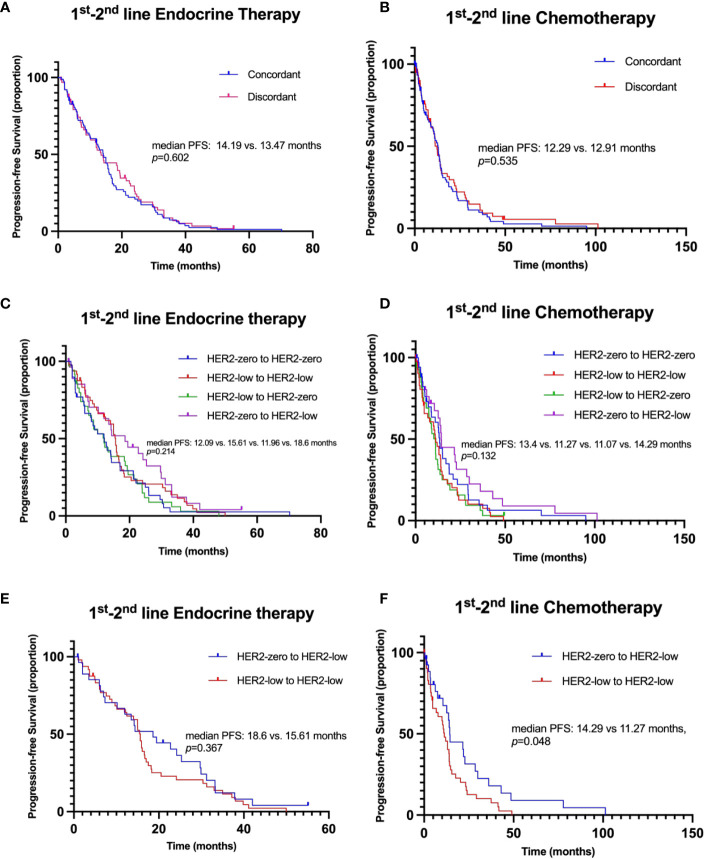
Kaplan–Meier curves for progression-free survival in the 1^st^-2^nd^ line systemic therapy. **(A)** Comparison of PFS in patients with concordant versus discordant HER2 status between primary breast cancer (BC) and recurrence/metastasis during 1^st^-2^nd^ line endocrine therapy. **(B)** Comparison of PFS in patients with concordant versus discordant HER2 status during 1^st^-2^nd^ line chemotherapy. **(C)** Analysis of PFS in patients categorized by changes in HER2 status from primary BC to recurrence/metastasis during 1st-2nd line endocrine therapy. **(D)** Analysis of PFS in patients categorized by changes in HER2 status from primary BC to recurrence/metastasis during 1^st^-2^nd^ line chemotherapy. **(E)** Comparison of PFS in patients with different HER2 status transitions (HER2-zero to HER2-low versus HER2-low to HER2-low) during 1^st^-2^nd^ line endocrine therapy. **(F)** Comparison of PFS in patients with different HER2 status transitions (HER2-zero to HER2-low vs. HER2-low to HER2-low) during 1^st^-2^nd^ line chemotherapy.

Additionally, concordance or discordance in HER2 status did not significantly impact PFS during 1st-2nd line chemotherapy (median PFS: 12.29 vs. 12.91 months, p=0.535, HR 1.119, 95% CI 0.785-1.593; **Figure 6B**). However, patients with a transition in HER2 status from zero to low displayed a significantly longer PFS compared to those who maintained a low status (median PFS: 14.29 vs 11.27 months, p=0.048, HR 0.597, 95% CI 0.358-0.996; **Figure 6F**).

#### Impact of HER2 status evolution on therapy efficacy

Interestingly, the evolution of HER2 status from primary BC to recurrence/metastasis displayed potential impacts on treatment outcomes. Variations were observed in PFS among different patterns of HER2 evolution during 1st-2nd line endocrine therapy; however, these differences did not reach statistical significance (HER2-zero to HER2-zero vs. HER2-low to HER2-low vs. HER2-low to HER2-zero vs. HER2-zero to HER2-low median PFS: 12.09 vs. 15.61 vs. 11.96 vs. 18.6 months, p=0.214; **Figure 6C**). This trend was mirrored in the 1st-2nd line chemotherapy setting, with the difference not being statistically significant (median PFS: 13.4 vs. 11.27 vs. 11.07 vs. 14.29 months p=0.132; **Figure 6D**). Importantly, echoing the above observation, patients transitioning in HER2 status from zero to low had a longer PFS than those remaining low during 1st-2nd line chemotherapy (median PFS: 14.29 vs. 11.27 months, p=0.048, HR 0.597, 95% CI 0.358-0.996; **Figure 6F**). However, this distinction in PFS was not observed during the 1st-2nd line of endocrine therapy (median PFS: 18.6 vs. 15.61 months, p=0.357, HR 1.192, 95% CI 0.731-1.943; **Figure 6E**).

## Discussion

In our cohort of 458 HoR-positive/HER2-negative breast cancer patients, we found that HER2-zero patients may respond better to chemotherapy than HER2-low patients. Furthermore, compared to patients who remained HER2-low, those who switched from HER2-zero to HER2-low were more likely to benefit from chemotherapy.

HER2-low breast cancer accounted for almost a half of the HoR-positive/HER2-negative cohort (54.37%), which is consistent with available evidence (3, 17, 18). When confined to patients with HoR-positive advanced breast cancer, our study revealed no significant difference in clinicopathological characteristics between the HER2-zero and HER2-low groups. Up to today, results of prior investigations are inconsistent, and the clinicopathological characteristics of HER2-low breast cancer are not yet completely understood. According to a pooled analysis of 2,310 HER2-negative breast cancer patients from four prospective neoadjuvant clinical trials (17), fewer grade III tumors, a lower Ki-67 status, and fewer TP53 mutations were found in the HER2-low patients. However, in another analysis (19), which was in metastatic situation, there were no appreciable differences in patient demographics or clinical features between HER2-low and HER2-zero patients. Various studies (17, 18, 20, 21) have also analyzed the HER2 status of patients with a distinct HoR background. In a prior investigation of the Chinese MBC population (18), in the HoR-positive subgroup, HER2-low tumors were associated with a larger proportion of stage IV cancer and fewer invasive lobular tumors. Numerous researchers (20) suggested that the discrepancy between HER2-zero and HER2-low baseline features contributed to the large disparity in HoR-positivity. It is unknown whether HER2-low breast cancer has more aggressive characteristics, given the contradictory findings of several studies (7, 17). Our results did not support the notion that HER2-low breast cancer was biologically distinct from HER2-zero breast cancer in the HoR-positive group.

In terms of HER2-low expression, our study reveals a considerable discrepancy between primary tumors and matched advanced-stage samples in the HoR-positive population, observing a 41.18% overall rate of HER2 discordance between primary and recurrent/metastatic samples (K=0.17, 95%CI 0.02-0.33). This result is consistent with the earlier research (22–24),which revealed the instability of HER2-low expression from primary to metastatic tumors. It is worth mentioning that earlier research (22) demonstrated the HoR-positive/HER2-negative group was mostly responsible for the HER2-low expression discordance from or to the HER2-zero phenotype. Significant discordance was also observed when restricting the cohort of patients with HoR-positive tumors in other researches (22–24).

It may be due to multiple reasons, including analytical parameters, HER2 expression heterogeneity and biological evolution of the disease. The variability of HER2 expression is predicted to be significantly influenced by technical factors associated with the HER2 testing methodologies (3). The determination of HER2 IHC scoring exhibits significant inter-observer variability. In a recent study (25), there was poor concordance between HER2 0 & HER2 1+ among 18 experienced pathologists. Moreover, discordance was seen within a patient with tissue from different locations at the same timepoint, even within one organ (26). This observation may worth consideration when dealing with HoR-positive/HER2-negative advanced breast cancer patients who have exhausted their primary treatment options, such as hormonal therapies and chemotherapy, but who may still benefit from additional treatments. Those with HER2-low expression may be good candidates for participation in ongoing clinical trials of anti-HER2 ADCs. Our findings imply that the evolution of HER2-low expression from primary to recurrent tumors within the HoR-positive breast cancer involves a non-negligible proportion of HER2-negative breast cancer patients, thus warranting further investigation.

This study’s primary purpose is to examine the response to different treatment strategies between HER2-low and HER2-zero groups in HoR-positive patients. As a results, we detected no significant difference in endocrine therapy between HoR-positive/HER2-negative individuals with different HER2 status (HER2-low *vs.* HER2-zero). This is similar with the findings of an MD Anderson Series research (27), which demonstrated no difference in PFS and OS between HER2-low and HER2-zero patients receiving ET+ CDK 4/6i in the first-line context. Tarantino P et. al (23) also performed a sub-analysis of 1^st^ line PFS in a research based on HER2-expression, in patients receiving first-line endocrine therapy (aromatase inhibitor or fulvestrant) plus a CDK4/6 inhibitor (palbociclib, ribociclib, or abemaciclib), and no significant difference in 1^st^ line PFS was found between HER2-low and HER2-zero tumors in this sub-analysis. Perhaps it can be explained by the gene expression profile investigations (7) demonstrating that there were few physiologic differences in HoR-positive BC based on the expression of HER2-low, except for overexpressed ERBB2 and luminal-related genes. Nevertheless, it should be noted that the opposite result was seen in some retrospective research. A publication of retrospective data (28) observed that HER2-low expression was associated with an inferior PFS among patients with HoR-positive/HER2-negative MBC treated with CDK4/6 inhibitors. Moreover, a multicenter Italian investigation (29) indicated that HER2-low status has independent negative prognostic value in patients with HoR-positive/HER-negative advanced BC treated with CDK4/6i with ET as first-line therapy. It has been known for a long time that HER2 amplification reduces anti-estrogen therapy sensitivity, principally through activating alternative survival pathways (30) (ie, PI3K-AKT and MAPK pathways). Data (30, 31) from preclinical and clinical studies suggest that HER2 may eventually assume the driving role in tumor progression by serving as an alternate survival pathway or by lowering the level of ER, so rendering the tumor less estrogen-sensitive. According to the studies mentioned above, it is still an unresolved issue, regarding the connection between HER2 low expression and the effectiveness of endocrine therapy. Given the possibility of bias in the retrospective study, additional research is warranted.

Our study reveals that HoR-positive/HER2-zero patients typically respond more favorably to chemotherapy compared to HoR-positive/HER2-low patients in the first and second-line setting. Intriguingly, HoR-positive participants whose HER2 status transitioned from zero to low exhibited longer PFS than those consistently presenting as HER2-low when undergoing first and second-line chemotherapy for HoR-positive MBC. Interestingly, previous reports have shown that while HER2 status doesn’t impact the pathological complete response (pCR) in HoR-negative patients, the HER2-low status correlates with a reduced pCR rate in HoR-positive patients within the neoadjuvant chemotherapy (NAC) setting (9, 17, 32). Multivariable analyses have further demonstrated that HER2-low status serves as an independent predictor of pCR in HoR-positive patients, whereas HoR-negative/HER2-low status does not seem to influence NAC efficacy (9). These findings suggest that HER2-low status might affect the efficacy of chemotherapy in luminal BC, a subtype typically resistant to conventional cytotoxics (32). This provides additional rationale for testing anti-HER2 ADCs in HER2-low/HoR-positive BC. The precise mechanism, however, remains to be fully elucidated. Given the current research landscape, it’s reasonable to suggest that HER2-low status may be linked to reduced chemotherapy efficacy in breast cancer patients in the first and second-line setting. Nevertheless, differing HoR statuses must also be taken into consideration.

Our study does present certain limitations. Firstly, it’s a retrospective analysis, which could introduce imbalance and referral bias among the groups. Secondly, some participants had their HER2-expression analyzed outside of our institution, and some biopsy samples were taken amidst anticancer treatments, which might affect HER2-expression results. Furthermore, the evolving criteria for HER2 status interpretation should be taken into account (4, 33). Thirdly, multiple factors that could affect PFS, including visit scheduling, patient adherence to therapy, and different evaluation cycles for treatment response. As such, outcomes should be viewed cautiously. Given these factors, further large-scale, prospective clinical studies are necessary to explore the features and responses of advanced HoR-positive/HER2-low breast cancer in greater depth.

## Conclusion

In HoR-positive MBC, patients with low or zero HER2 expression show similar responses to endocrine treatment, but those with low HER2 expression have worse chemotherapy outcomes. Changes in HER2 status could affect chemotherapy results, hence HER2 levels and heterogeneity are important treatment considerations.

## Data availability statement

The raw data supporting the conclusions of this article will be made available by the authors, without undue reservation.

## Ethics statement

The studies involving humans were approved by the Institutional Review Board of Fudan University Cancer Hospital (SCCIRB). The studies were conducted in accordance with the local legislation and institutional requirements. The ethics committee/institutional review board waived the requirement of written informed consent for participation from the participants or the participants’ legal guardians/next of kin because the study is a retrospective investigation.

## Author contributions

SY: Data curation, Formal Analysis, Methodology, Project administration, Writing – original draft. CG: Data curation, Writing – review & editing. YiL: Funding acquisition, Data curation, Writing – review & editing. YX: Writing – review & editing, Data curation. YuL: Data curation, Writing – review & editing. YZ: Writing – original draft, Conceptualization, Formal Analysis, Funding acquisition, Supervision, Writing – review & editing. BW: Conceptualization, Supervision, Writing – review & editing, Funding acquisition.

## References

[1] Siegel RL Miller KD Fuchs HE Jemal A. Cancer statistics, 2022. CA Cancer J Clin (2022) 72(1):7–33. doi: 10.3322/caac.21708 35020204

[2] Arnold M Morgan E Rumgay H Mafra A Singh D Laversanne M. Current and future burden of breast cancer: Global statistics for 2020 and 2040. Breast (2022) 66:15–23. doi: 10.1016/j.breast.2022.08.010 36084384 PMC9465273

[3] Tarantino P Hamilton E Tolaney SM Cortes J Morganti S Ferraro E. HER2-low breast cancer: pathological and clinical landscape. J Clin Oncol (2020) 38(17):1951–62. doi: 10.1200/JCO.19.02488 32330069

[4] Wolff AC Hammond MEH Allison KH Harvey BE Mangu PB Bartlett JMS. Human epidermal growth factor receptor 2 testing in breast cancer: American society of clinical oncology/college of American pathologists clinical practice guideline focused update. J Clin Oncol (2018) 36(20):2105–22. doi: 10.1200/JCO.2018.77.8738 29846122

[5] Prat A Bardia A Curigliano G Hammond MEH Loibl S Tolaney SM. An overview of clinical development of agents for metastatic or advanced breast cancer without ERBB2 amplification (HER2-low). JAMA Oncol (2022) 8(11):1676–87. doi: 10.1001/jamaoncol.2022.4175 36107417

[6] Modi S Jacot W Yamashita T Sohn J Vidal M Tokunaga E. Trastuzumab deruxtecan in previously treated HER2-low advanced breast cancer. N Engl J Med (2022) 387(1):9–20. doi: 10.1056/NEJMoa2203690 35665782 PMC10561652

[7] Schettini F Chic N Brasó-Maristany F Paré L Pascual T Conte B. Clinical, pathological, and PAM50 gene expression features of HER2-low breast cancer. NPJ Breast Cancer (2021) 7(1):1. doi: 10.1038/s41523-020-00208-2 33397968 PMC7782714

[8] Agostinetto E Rediti M Fimereli D Debien V Piccart M Aftimos P. HER2-low breast cancer: molecular characteristics and prognosis. Cancers (Basel) (2021) 13(11):2824. doi: 10.3390/cancers13112824 34198891 PMC8201345

[9] Shao Y Yu Y Luo Z Guan H Zhu F He Y. Clinical, pathological complete response, and prognosis characteristics of HER2-low breast cancer in the neoadjuvant chemotherapy setting: A retrospective analysis. Ann Surg Oncol (2022) 29(13):8026–34. doi: 10.1245/s10434-022-12369-4 35933542

[10] Tarantino P Jin Q Tayob N Jeselsohn RM Schnitt SJ Vincuilla J. Prognostic and biologic significance of ERBB2-low expression in early-stage breast cancer. JAMA Oncol (2022) 8(8):1177–83. doi: 10.1001/jamaoncol.2022.2286 PMC9227690 35737367

[11] Jacot W Maran-Gonzalez A Massol O Sorbs C Mollevi C Guiu S. Prognostic value of HER2-low expression in non-metastatic triple-negative breast cancer and correlation with other biomarkers. Cancers (Basel) (2021) 13(23):6059. doi: 10.3390/cancers13236059 34885167 PMC8656488

[12] Osborne CK Schiff R. Mechanisms of endocrine resistance in breast cancer. Annu Rev Med (2011) 62(1):233–47. doi: 10.1146/annurev-med-070909-182917 PMC3656649 20887199

[13] Mutai R Barkan T Moore A Sarfaty M Shochat T Yerushalmi R. Prognostic impact of HER2-low expression in hormone receptor positive early breast cancer. Breast (2021) 60:62–9. doi: 10.1016/j.breast.2021.08.016 PMC8414540 34481367

[14] Hein A Hartkopf AD Emons J Lux MP Volz B Taran FA. Prognostic effect of low-level HER2 expression in patients with clinically negative HER2 status. Eur J Cancer (2021) 155:1–12. doi: 10.1016/j.ejca.2021.06.033 34311211

[15] Cardoso F Paluch-Shimon S Senkus E Curigliano G Aapro MS André F. 5th ESO-ESMO international consensus guidelines for advanced breast cancer (ABC 5). Ann Oncol (2020) 31(12):1623–49. doi: 10.1016/j.annonc.2020.09.010 PMC7510449 32979513

[16] Cardoso F Senkus E Costa A Papadopoulos E Aapro M André F. 4th ESO-ESMO international consensus guidelines for advanced breast cancer (ABC 4)†. Ann Oncol (2018) 29(8):1634–57. doi: 10.1093/annonc/mdy192 PMC7360146 30032243

[17] Denkert C Seither F Schneeweiss A Link T Blohmer JU Just M. Clinical and molecular characteristics of HER2-low-positive breast cancer: pooled analysis of individual patient data from four prospective, neoadjuvant clinical trials. Lancet Oncol (2021) 22(8):1151–61. doi: 10.1016/S1470-2045(21)00301-6 34252375

[18] Li Y Abudureheiyimu N Mo H Guan X Lin S Wang Z. In real life, low-level HER2 expression may be associated with better outcome in HER2-negative breast cancer: A study of the national cancer center, China. Front Oncol (2021) 11:774577. doi: 10.3389/fonc.2021.774577 35111669 PMC8801428

[19] Viale G Basik M Niikura N Tokunaga E Brucker S Penault-Llorca F. Retrospective study to estimate the prevalence and describe the clinicopathological characteristics, treatments received, and outcomes of HER2-low breast cancer. ESMO Open (2023) 8(4):101615. doi: 10.1016/j.esmoop.2023.101615 37562195 PMC10515285

[20] Xu H Han Y Wu Y Wang Y Li Q Zhang P. Clinicopathological characteristics and prognosis of HER2-low early-stage breast cancer: A single-institution experience. Front Oncol (2022) 12:906011. doi: 10.3389/fonc.2022.906011 35785207 PMC9245921

[21] Horisawa N Adachi Y Takatsuka D Nozawa K Endo Y Ozaki Y. The frequency of low HER2 expression in breast cancer and a comparison of prognosis between patients with HER2-low and HER2-negative breast cancer by HR status. Breast Cancer (2022) 29(2):234–41. doi: 10.1007/s12282-021-01303-3 34622383

[22] Miglietta F Griguolo G Bottosso M Giarratano T Lo Mele M Fassan M. Evolution of HER2-low expression from primary to recurrent breast cancer. NPJ Breast Cancer (2021) 7(1):137. doi: 10.1038/s41523-021-00343-4 34642348 PMC8511010

[23] Tarantino P Gandini S Nicolò E Trillo P Giugliano F Zagami P. Evolution of low HER2 expression between early and advanced-stage breast cancer. Eur J Cancer (2022) 163:35–43. doi: 10.1016/j.ejca.2021.12.022 35032815

[24] Miglietta F Griguolo G Bottosso M Giarratano T Lo Mele M Fassan M. HER2-low-positive breast cancer: evolution from primary tumor to residual disease after neoadjuvant treatment. NPJ Breast Cancer (2022) 8(1):66. doi: 10.1038/s41523-022-00434-w 35595761 PMC9122970

[25] Fernandez AI Liu M Bellizzi A Brock J Fadare O Hanley K. Examination of low ERBB2 protein expression in breast cancer tissue. JAMA Oncol (2022) 8(4):1–4. doi: 10.1001/jamaoncol.2021.7239 PMC8814969 35113160

[26] Geukens T De Schepper M Richard F Maetens M Van Baelen K Mahdami A. Intra-patient and inter-metastasis heterogeneity of HER2-low status in metastatic breast cancer. Eur J Cancer (2023) 188:152–60. doi:10.1016/j.ejca.2023.04.026 37247580

[27] Mouabbi JA Raghavendra AS Bassett RL Hassan A Tripathy D Layman RM. Histology-based survival outcomes in hormone receptor-positive metastatic breast cancer treated with targeted therapies. NPJ Breast Cancer (2022) 8:131. doi: 10.1038/s41523-022-00499-7 36539444 PMC9768132

[28] Bao KKH Sutanto L Tse SSW Man Cheung K Chan JCH. The association of ERBB2-low expression with the efficacy of cyclin-dependent kinase 4/6 inhibitor in hormone receptor-positive, ERBB2-negative metastatic breast cancer. JAMA Netw Open (2021) 4(11):e2133132. doi: 10.1001/jamanetworkopen.2021.33132 34739066 PMC8571658

[29] Zattarin E Presti D Mariani L Sposetti C Leporati R Menichetti A. Prognostic significance of HER2-low status in HR-positive/HER2-negative advanced breast cancer treated with CDK4/6 inhibitors. NPJ Breast Cancer (2023) 9(1):27. doi: 10.1038/s41523-023-00534-1 37069173 PMC10110597

[30] Arpino G Green SJ Allred DC Lew D Martino S Osborne CK. HER-2 amplification, HER-1 expression, and tamoxifen response in estrogen receptor-positive metastatic breast cancer: a southwest oncology group study. Clin Cancer Res (2004) 10(17):5670–6. doi: 10.1158/1078-0432.CCR-04-0110 15355892

[31] Lopez-Tarruella S Schiff R. The dynamics of estrogen receptor status in breast cancer: re-shaping the paradigm. Clin Cancer Res (2007) 13(23):6921–5. doi: 10.1158/1078-0432.CCR-07-1399 18056165

[32] de Nonneville A Houvenaeghel G Cohen M Sabiani L Bannier M Viret F. Pathological complete response rate and disease-free survival after neoadjuvant chemotherapy in patients with HER2-low and HER2-0 breast cancers. Eur J Cancer (2022) 176:181–8. doi: 10.1016/j.ejca.2022.09.017 36257173

[33] Wolff AC Hammond MEH Hicks DG Dowsett M McShane LM Allison KH. Recommendations for human epidermal growth factor receptor 2 testing in breast cancer: American Society of Clinical Oncology/College of American Pathologists clinical practice guideline update. J Clin Oncol (2013) 31(31):3997–4013. doi: 10.1200/JCO.2013.50.9984 24101045

