# Supplementary material for: Clinicopathological characteristics, evolution, treatment pattern and outcomes of hormone-receptor-positive/HER2-low metastatic breast cancer
Source: Front Endocrinol (Lausanne). 2023 Oct 10;14:1270453. doi: 10.3389/fendo.2023.1270453 (PMC10595148; doi:10.3389/fendo.2023.1270453)
Supplement: Supplementary file 1 [file Image_1.pdf]

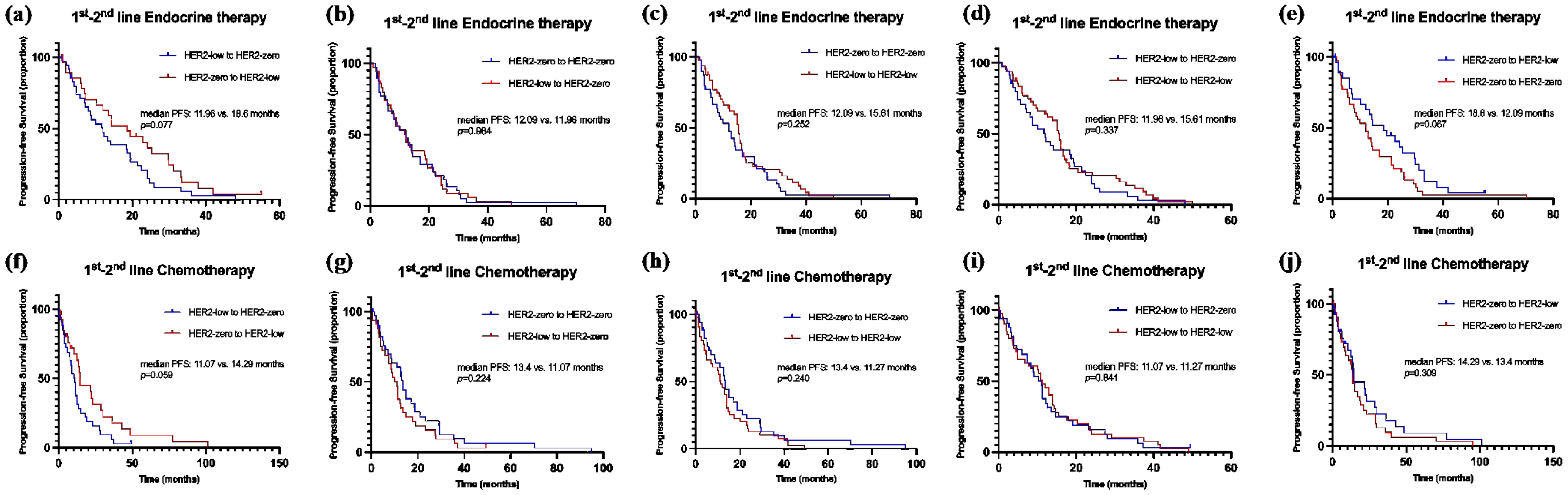

Supplementary Figure 1. Kaplan-Meier analysis of progression-free survival (PFS) during 1st-2nd line systemic therapy stratified by changes in HER2 status from primary to recurrent/metastatic breast cancer (BC). The comparisons are as follows: (a) PFS in patients receiving 1st-2nd line endocrine therapy with changes from HER2-low to HER2-zero vs. HER2-zero to HER2-low. (b) PFS in patients receiving 1st-2nd line endocrine therapy with changes from HER2-zero to HER2-zero vs. HER2-low to HER2-zero. (c) PFS in patients undergoing the same therapy with changes from HER2-zero to HER2-zero vs. HER2-low to HER2-low. (d) PFS in patients undergoing the same therapy with changes from HER2-low to HER2-zero vs. HER2-low to HER2-low respectively. (e) PFS in patients receiving 1st-2nd line endocrine therapy with changes from HER2-zero to HER2-low vs. HER2-zero to HER2-zero. (f) PFS in patients receiving 1st-2nd line chemotherapy with changes from HER2-low to HER2-zero vs. HER2-zero to HER2-low. (g) PFS in patients receiving 1st-2nd line chemotherapy with changes from HER2-zero to HER2-zero vs. HER2-low to HER2-zero (h) PFS in patients undergoing the same chemotherapy with changes from HER2-zero to HER2-zero vs. HER2-low to HER2-low. (i) PFS in patients undergoing the same chemotherapy with changes from HER2-low to HER2-zero vs. HER2-low to HER2-low. (j) PFS in patients receiving 1st-2nd line chemotherapy with changes from HER2-zero to HER2-low vs. HER2-zero to HER2-zero.

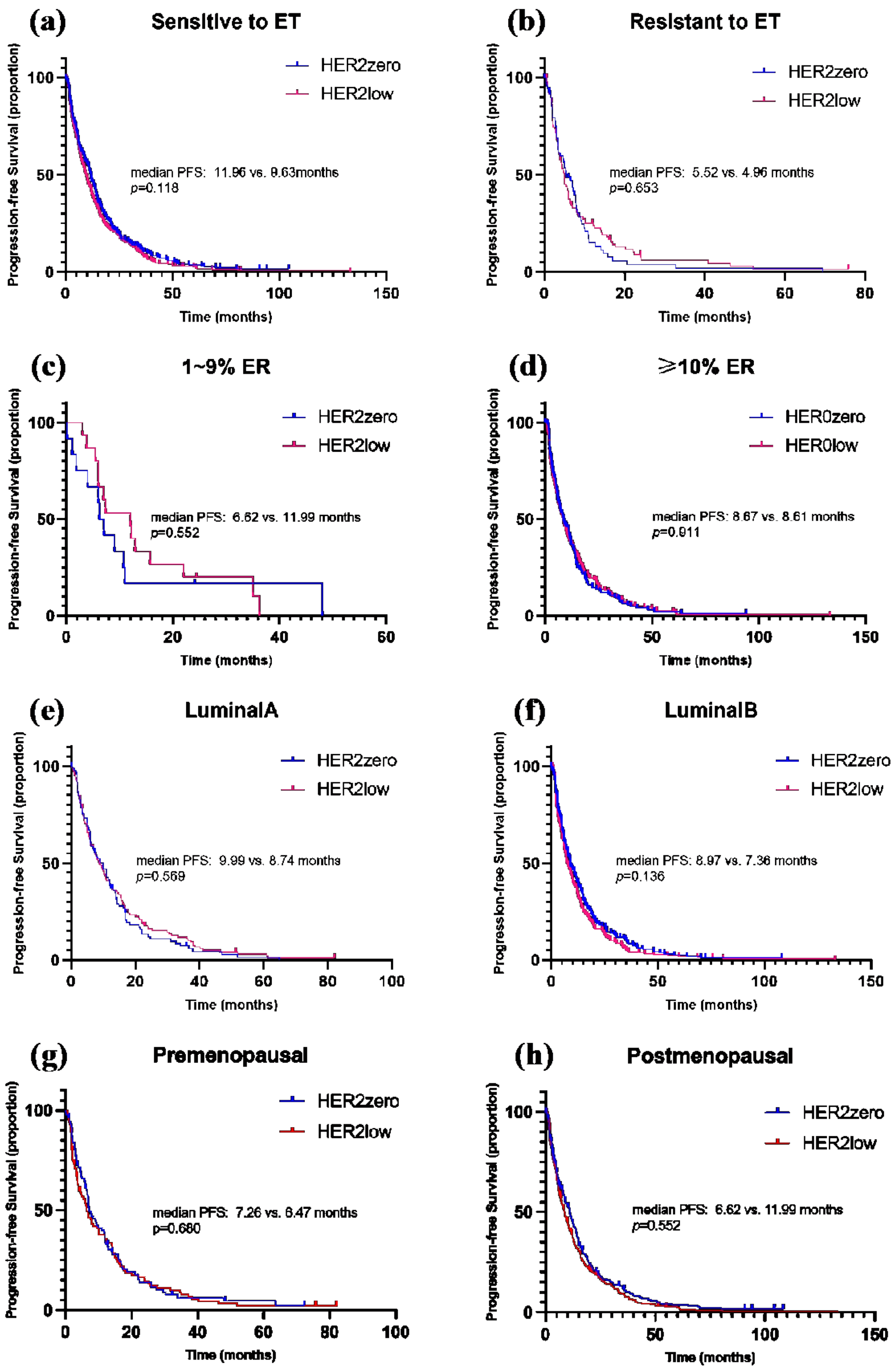

Supplementary Figure 2. Kaplan–Meier curves for progression-free survival (PFS) in the 1st and 2nd line systemic endocrine therapy (ET) by HER2 status in the different stratified population. PFS for HER2-zero vs. HER2-low patients in the 1st and 2nd line systemic therapy for the (a) ET-sensitive population, (b) ET-resistant population, (c) 1~9% ER population, (d) ≥10% ER population, (e) Luminal A population, (f) Luminal B population, (g) premenopausal population, (h) postmenopausal population. *p* values are from the log-rank test. ET, endocrine therapy.
